# 1-[(Anthracen-9-yl)carbon­yl]-2,7-di­meth­oxy­naphthalene: a chain-like structure composed of face-to-face type dimeric mol­ecular aggregates

**DOI:** 10.1107/S2056989016018077

**Published:** 2016-11-18

**Authors:** Siqingaowa, Takehiro Tsumuki, Kazuki Ogata, Noriyuki Yonezawa, Akiko Okamoto

**Affiliations:** a Department of Organic and Polymer Materials Chemistry, Tokyo University of Agriculture & Technology (TUAT), Koganei, Tokyo 184-8588, Japan

**Keywords:** crystal structure, independent mol­ecules, face-to-face type dimeric mol­ecular aggregate, complementary hydrogen bonds, non-coplanarly accumulated aromatic rings arrangement

## Abstract

The asymmetric unit of the title compound, C_27_H_20_O_3_, contains two independent mol­ecules (*A* and *B*). The anthracene ring system is connected to the 2,7-di­meth­oxy­naphthalene core in a twisted manner, with dihedral angles of 86.38 (5) and 79.36 (8)°, respectively, for conformers *A* and *B*. In the crystal, face-to-face type dimeric mol­ecular aggregates of each conformer are observed. The dimer of conformer *A* is formed by two pairs of C—H⋯π inter­actions, and that of conformer *B* by a pair of (*sp* ^2^)C—H⋯O hydrogen bonds. The dimers of conformer *A* are linked to each other *via* a π–π stacking inter­action between the anthracene rings to form a chain along the *b* axis and the chains are aligned along the *c* axis, forming a sheet structure. The dimers of conformer *B* are connected to each other *via* a couple of C—H⋯π inter­actions to form a chain along the *b* axis. The chains are aligned along the *c* axis through (*sp* ^2^)C—H⋯O=C hydrogen bonds, forming a sheet parallel to the *bc* plane. The sheets of conformers *A* and *B* are alternately stacked along the *a* axis *via* two kinds of inter­molecular (*sp* ^2^)C—H⋯O=C hydrogen bonds.

## Abstract 2

The asymmetric unit of the title compound contains two independent mol­ecules (*A* and *B*). In the crystal, mol­ecules of each conformer make a face-to-face type dimeric mol­ecular aggregate with two pairs of C—H⋯π hydrogen bonds (*A*) or a pair of (*sp* ^2^)C—H⋯O hydrogen bonds (*B*). The dimeric mol­ecular aggregates composed of same conformers are linked to each other into a chain through π–π stacking inter­actions (*A*) or a pair of C—H⋯π hydrogen bonds (*B*) along the *b* axis. The chains are aligned along the *c* axis by weak van der Waals inter­actions or (*sp* ^2^)C—H⋯O=O hydrogen bonds, and they are alternately stacked along the *a* axis.

## Chemical context   

Compounds of coplanar aggregation of π-conjugated aromatic rings have received attention from a wide range of material chemists and organic ones because of their excellent conductivity properties (Lu *et al.*, 2010 ▸). Recently, uniquely shaped π-conjugated aromatic aggregation compounds have moved into the limelight as promising mol­ecular frameworks in nano­electronics, *e.g*. bucky bowls (Schmidt *et al.*, 2013 ▸), coannulene (Yoshimoto *et al.*, 2010 ▸) and cyclo­para­phenyl­ene (Bunz *et al.*, 2012 ▸). These compounds can be regarded as mol­ecules of partial structure and motif of fullerene and carbon nanotubes. On the other hand, aromatic aggregate compounds bearing a *non-consecutive* π-conjugated structure have also started to garner attention. For example, the mol­ecular geometry of 9-aryl­anthracene compounds is of photochemical and photophysical inter­est because a coplanar spatial arrangement of the anthracene and the aryl substituent π-systems is precluded due to intra­molecular hindrance involving the hydrogen atoms (Becker *et al.*, 1992 ▸). In such mol­ecules, the π-conjugation is weakened and deviations from mol­ecular planarity are borne out in electronic absorption and emission spectra. In partic­ular, the fluorescence spectra of non-coplanarly situated bichromophoric compounds, characterized by large Stokes shifts, are indicative of differences between the geometry of the ground state and that of the more planar emitting excited state (Becker *et al.*, 1990 ▸).
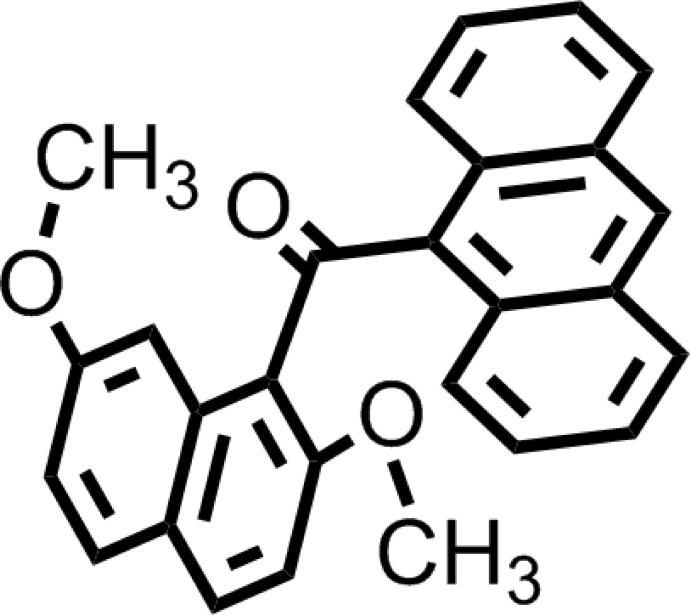


The present authors have studied the synthesis and structure analysis of *peri*(1,8)-aroylated naphthalene compounds in which aromatic rings accumulate non-coplanarly, giving highly congested intra­molecular circumstances (Okamoto & Yonezawa, 2015 ▸; Okamoto *et al.*, 2016 ▸). As one of the categories of the accumulated π-conjugated aromatic ring compounds, *peri*-aroyl­naphthalene compounds have some distinguishable structural characteristics. *peri*-Aroyl­naphthalene compounds belong to the class of poly(aromatic ring) mol­ecules where aromatic rings are linked by ketonic carbonyl groups. Furthermore, the two aroyl groups at *peri*-positions of the naphthalene ring core are situated in positions very close to each other. Accordingly, a coplanar alignment of the aromatic rings in a mol­ecule is not possible in *peri*-aroyl­naphthalene compounds because of their highly congested mol­ecular arrangement. On the other hand, the spatial organization around the ketonic carbonyl groups of a diaryl ketone structure is supposed to be rather loose compared to that of directly combined aromatic ring systems, as shown in the rotation barrier for an analogous compound in solution (Okamoto *et al.*, 2012 ▸). In this regard, the expected flexibility of the aromatic ketone compound probably shows great variation in the mol­ecular and packing structures in the crystal. Such a situation offers a good opportunity to reveal the hitherto unknown inter­actions that determine the structure of aromatic rings of accumulated mol­ecules in the crystalline state. This article reports the synthesis and crystal structure of the title 1-anthroylated naphthalene compound.

## Structural commentary   

**Figure 1:**
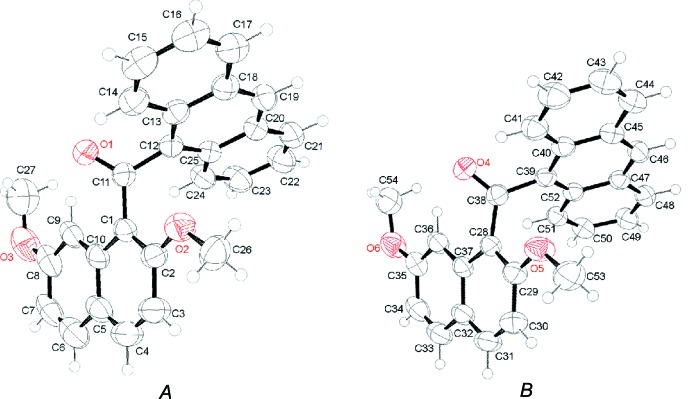
The structure of the independent mol­ecules *A* and *B*, showing the atom-labelling scheme. Displacement ellipsoids are drawn at the 50% probability level for non-H atoms.

There are two independent mol­ecules in the asymmetric unit of the title compound. The conformers, labeled *A* and *B*, are shown in Fig. 1 ▸. Each conformer has essentially the same non-coplanar structure, with the meth­oxy group adjacent to the anthroyl group being oriented to the *endo* side against the naphthalene ring core. The main difference between the conformers is in the orientation of the anthracene ring with respect to the naphthalene ring core. Conformer *A* shows a dihedral angle of 86.38 (5)° between the anthracene and naphthalene ring systems, which is slightly larger than that of 79.36 (8)° for conformer *B*.

## Supra­molecular features   

**Figure 2:**
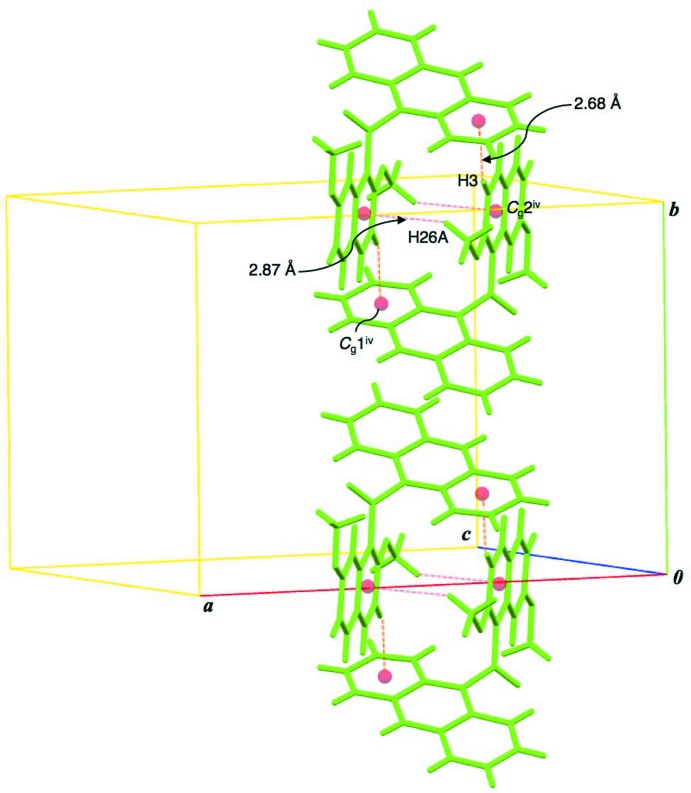
Dimeric mol­ecular aggregates of conformer *A*. Two types of complementary C—H⋯π inter­actions are shown as dashed lines. [Symmetry code: (iv) −*x* + 1, −*y* + 2, −*z*.]

**Figure 3:**
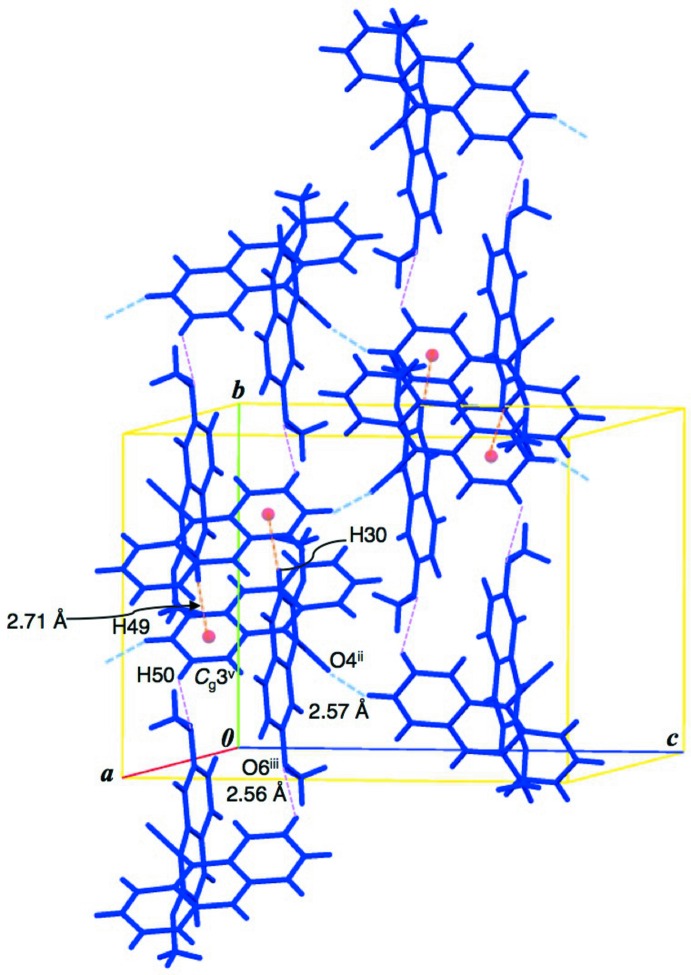
Dimeric mol­ecular aggregates of conformer *B* and the chain-like mol­ecular alignments. Two kinds of complementary C—H⋯π inter­actions and (*sp* ^2^)C—H⋯OMe hydrogen bonds are shown as orange and pink dashed lines. C—H⋯O=C hydrogen bonds between the chains are expressed as blue dashed lines. [Symmetry codes: (ii) *x*, −*y* + 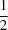, *z* − 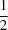; (iii) −*x*, −*y*, -*z;* (v) −*x*, −*y* + 1, −*z*.]

**Figure 4:**
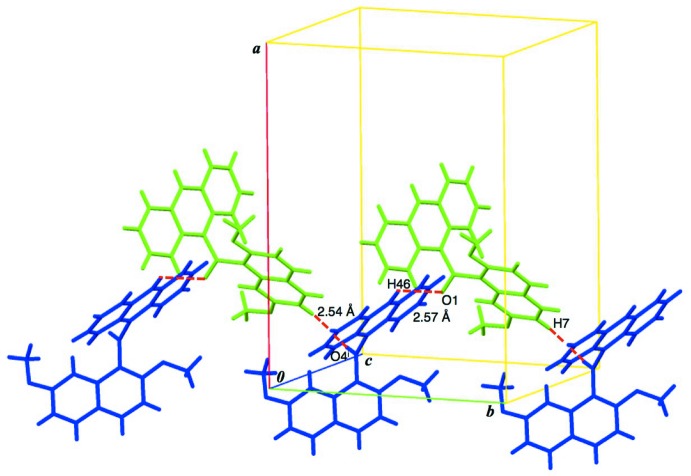
Two kinds of (*sp* ^2^)C—H⋯O=C hydrogen bonds between conformers *A* and *B* are shown as red broken lines. [Symmetry code: (i) *x*, *y* − 1, *z*.]

Observed non-covalent bonding inter­actions are summarized in Table 1 ▸. In the crystal structure, each conformer forms an inversion dimer with a face-to-face type mol­ecular aggregate by complementary hydrogen bonds. In the dimer of conformer *A*, a pair of (naphthalene)C—H⋯π (anthracene) inter­actions and another pair of (meth­oxy)C—H⋯π (naphthalene) ones are observed (C3—H3⋯*Cg*1^iv^ and C26—H26*A*⋯*Cg*2^iv^; symmetry code in Table 1 ▸; Fig. 2 ▸). The dimeric aggregates of conformers *A* are connected into a chain along the *b* axis through a π–π stacking inter­action between the anthracene rings [centroid-centroid distance of 3.8198 (10) Å between the C12–C13/C18–C20/C25 and C13–C18 rings]. The chains of conformer *A* are aligned along the *c* axis by weak van der Waals inter­actions, forming a sheet structure parallel to the *bc* plane. In the dimer of conformer *B*, a pair of (anthracene)C—H⋯O(meth­oxy) hydrogen bonds are observed (C50—H50⋯O6^iii^; Table 1 ▸ and Fig. 3 ▸). Furthermore, a pair of (naphthalene)C—H⋯π(anthracene) inter­actions connect the dimeric aggregates into a chain along the *b* axis (C30—H30⋯*Cg*3^v^; Table 1 ▸ and Fig. 3 ▸). The chains of conformer *B* are linked by inter­molecular (anthracene)C—H⋯O=C hydrogen bonds (C49—H49⋯O4^ii^; Table 1 ▸) along the *c* axis, forming a sheet parallel to the *bc* plane. The two sheets of conformers *A* and *B* are stacked alternately along the *a* axis by (naphthalene)C—H⋯O=C and (anthracene)C—H⋯O=C hydrogen bonds (C46—H46⋯O1 and C7—H7⋯O4^i^; Table 1 ▸ and Figs. 4 ▸ and 5 ▸).

## Database survey   

A search of the Cambridge Structural Database (CSD Version 5.37, update 2 February 2016; Groom *et al.*, 2016 ▸) showed 278 structures of 1-substituted naphthalene compounds containing 1-benzoyl­ated 2,7-di­alk­oxy­naphthalene and 1-naphtho­ylated 2,7-di­alk­oxy­naphthalene (including α-naphtho­ylated and β-naphtho­ylated homologues). The title compound is closely related to (2,7-di­meth­oxy­naphthalen-1-yl)(naphthalen-1-yl)methanone, (I) (Tsumuki *et al.*, 2013 ▸), (2,7-di­meth­oxy­naph­thalen-1-yl)(phen­yl)methanone, (II) (Kato *et al.*, 2010 ▸), and 2,7-dimeth­oxy-1-(2-naphtho­yl)naphthalene, (III) (Tsumuki *et al.*, 2012 ▸). These homologues have two, three and one independent mol­ecules, respectively, in the asymmetric units of (I), (II) and (III). The dihedral angles between the aromatic ring of the aroyl group and the 2,7-di­meth­oxy­naphthalene ring core are each 79.07 (4) and 88.19 (4)° for homologue (I), 75.34 (7), 86.47 (7) and 76.55 (6)° for homologue (II), and 80.44 (4)° for homologue (III), which are close to those in the title compound [79.36 (8) and 86.38 (5)°].

## Synthesis and crystallization   

To a solution of 9-anthroyl chloride (7.8 mmol, 1.88 g) and CH_2_Cl_2_ (9.0 mL), AlCl_3_ (7.8 mmol, 1.04 g) was gradually added. After stirring for 10 min, 2,7-di­meth­oxy­naphthalene (3.6 mmol, 0.67 g) was added to the CH_2_Cl_2_ solution. The reaction mixture was stirred in ice-bath for 6 h, then poured into ice-cold water. The aqueous layer was extracted with chloro­form (30 ml × 3) and the combined extracts were washed with 2 *M* aqueous NaOH solution (30 ml × 3) followed by washing with brine (20 ml × 3). The organic layer thus obtained was dried over anhydrous MgSO_4_. The solvent was removed under reduced pressure to give a cake. The title compound was separated from the crude product by column chromatography (eluent: toluene); isolated yield 42%. Yellow needle single crystals suitable for X-ray diffraction were obtained by repeated crystallization from ethyl acetate solution.

^1^H NMR (500 MHz, CDCl_3_) δ: 3.00 (3H, *s*), 3.86 (3H, *s*), 6.90 (1H, *d*, *J* = 9.0 Hz), 7.14 (1H, *dd*, *J* = 2.5, 9.0 Hz), 7.42 (2H, *dt*, *J* = 2.0, 7.0 Hz), 7.45 (2H, *dt*, *J* = 2.0, 7.0 Hz), 7.76 (1H, *d*, *J* = 9.0 Hz), 7.87 (1H, *d*, *J* = 9.0 Hz), 8.0–8.02 (4H, *m*), 8.12 (1H, *d*, *J* = 2.0 Hz), 8.49 (1H, *s*) p.p.m. ^13^C NMR (125 MHz, CDCl_3_) δ: 55.281, 56.178, 102.94, 111.11, 117.38, 124.07, 124.88, 125.08, 125.46, 126.15, 128.44, 128.51, 130.14, 131,11, 133.42, 134.32, 138.67, 138.76, 159.36, 160.43, 201.25 p.p.m. IR (KBr): 1640(C=O), 1618(Ar), 1511(Ar), 1250(OMe) cm^−1^. HRMS (*m*/*z*): [*M* + H]^+^ calculated for C_27_H_21_O_3_, 393.1491, found, 393.1494. m.p. = 444.8–446.9 K.

## Refinement   

Crystal data, data collection and structure refinement details are summarized in Table 2 ▸. All H atoms were found in a difference map and were subsequently refined as riding atoms, with C—H = 0.95 (aromatic) and 0.98 (meth­yl) Å, and with *U*_iso_(H) = 1.2 *U*_eq_(C). Rigid bond restraints (*DELU*) were applied to O3—C8, C1—C2, C2—C3, C4—C5, C5—C6, C7—C8 and C9—C10 in the naphthalene moiety.

## Supplementary Material

Crystal structure: contains datablock(s) I. DOI: 10.1107/S2056989016018077/is5460sup1.cif

Structure factors: contains datablock(s) I. DOI: 10.1107/S2056989016018077/is5460Isup2.hkl

ORTEP of molecule A for Fig 1. DOI: 10.1107/S2056989016018077/is5460sup3.ps

ORTEP of molecule B for Fig 1. DOI: 10.1107/S2056989016018077/is5460sup4.ps

Supporting information file. DOI: 10.1107/S2056989016018077/is5460Isup5.cml

CCDC reference: 1516088

Additional supporting information: crystallographic information; 3D view; checkCIF report

## Figures and Tables

**Figure 5:**
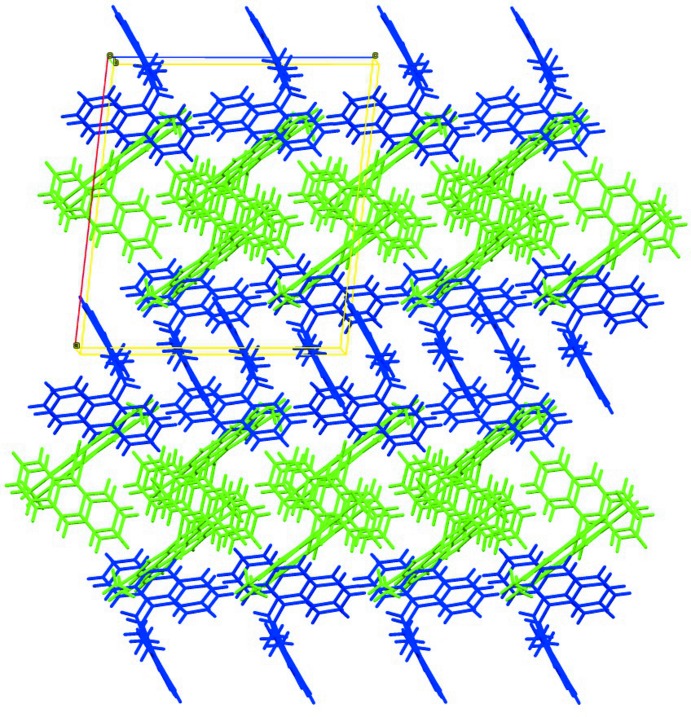
The arrangement of the mol­ecules in the crystal structure, viewed down the *b* axis.

**Table 1:** Hydrogen-bond geometry (Å, °) *Cg*1, *Cg*2 and *Cg*3 are the centroids of the C20–C25, C1–C5/C10 and C47–C52 rings, respectively.

*D*—H⋯*A*	*D*—H	H⋯*A*	*D*⋯*A*	*D*—H⋯*A*
C7—H7⋯O4^i^	0.95	2.54	3.419 (3)	154
C46—H46⋯O1	0.95	2.57	3.2925 (19)	133
C49—H49⋯O4^ii^	0.95	2.57	3.2515 (19)	128
C50—H50⋯O6^iii^	0.95	2.56	3.368 (2)	143
C3—H3⋯*Cg*1^iv^	0.95	2.68	3.557 (2)	153
C26—H26*A*⋯*Cg*2^iv^	0.98	2.87	3.730 (2)	147
C30—H30⋯*Cg*3^v^	0.95	2.71	3.602 (2)	157

Symmetry codes: (i) 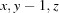; (ii) 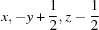; (iii) 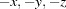; (iv) 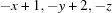; (v) 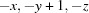.

**Table 2:** Experimental details

Crystal data	
Chemical formula	C_27_H_20_O_3_
*M*_r_	392.43
Crystal system, space group	Monoclinic, *P*2_1_/*c*
Temperature (K)	193
*a*, *b*, *c* (Å)	18.5975 (3), 12.9604 (2), 16.8900 (3)
β (°)	96.3650 (7)
*V* (Å^3^)	4045.92 (12)
*Z*	8
Radiation type	Cu *K*α
μ (mm^−1^)	0.66
Crystal size (mm)	0.60 × 0.30 × 0.10

Data collection	
Diffractometer	Rigaku R-AXIS RAPID
Absorption correction	Numerical (*NUMABS*; Higashi, 1999 ▸)
*T*_min_, *T*_max_	0.692, 0.937
No. of measured, independent and observed [*I* > 2σ(*I*)] reflections	59215, 7401, 4629
*R*_int_	0.070
(sin θ/λ)_max_ (Å^−1^)	0.602

Refinement	
*R*[*F* ^2^ > 2σ(*F* ^2^)], *wR*(*F* ^2^), *S*	0.040, 0.113, 0.97
No. of reflections	7401
No. of parameters	546
No. of restraints	7
H-atom treatment	H-atom parameters constrained
Δρ_max_, Δρ_min_ (e Å^−3^)	0.31, −0.22

Computer programs: *PROCESS-AUTO* (Rigaku, 1998 ▸), *CrystalStructure* (Rigaku, 2010 ▸), *Il Milione* (Burla *et al.*, 2007 ▸), *SHELXL97* (Sheldrick, 2008 ▸) and *ORTEPIII* (Burnett & Johnson, 1996 ▸).

## supplementary crystallographic information

### Crystal data

**Table:**

C_27_H_20_O_3_	*F*(000) = 1648
*M**_r_* = 392.43	*D*_x_ = 1.289 Mg m^−^^3^
Monoclinic, *P*2_1_/*c*	Melting point = 444.8–446.9 K
Hall symbol: -P 2ybc	Cu *K*α radiation, λ = 1.54187 Å
*a* = 18.5975 (3) Å	Cell parameters from 45896 reflections
*b* = 12.9604 (2) Å	θ = 3.4–68.2°
*c* = 16.8900 (3) Å	µ = 0.66 mm^−^^1^
β = 96.3650 (7)°	*T* = 193 K
*V* = 4045.92 (12) Å^3^	Platelet, yellow
*Z* = 8	0.60 × 0.30 × 0.10 mm

### Data collection

**Table:**

Rigaku R-AXIS RAPID diffractometer	7401 independent reflections
Radiation source: rotating anode	4629 reflections with *I* > 2σ(*I*)
Graphite monochromator	*R*_int_ = 0.070
Detector resolution: 10.00 pixels mm^-1^	θ_max_ = 68.2°, θ_min_ = 4.2°
ω scans	*h* = −22→22
Absorption correction: numerical (*NUMABS*; Higashi, 1999)	*k* = −15→15
*T*_min_ = 0.692, *T*_max_ = 0.937	*l* = −20→20
59215 measured reflections	

### Refinement

**Table:**

Refinement on *F*^2^	Secondary atom site location: difference Fourier map
Least-squares matrix: full	Hydrogen site location: inferred from neighbouring sites
*R*[*F*^2^ > 2σ(*F*^2^)] = 0.040	H-atom parameters constrained
*wR*(*F*^2^) = 0.113	*w* = 1/[σ^2^(*F*_o_^2^) + (0.0601*P*)^2^] where *P* = (*F*_o_^2^ + 2*F*_c_^2^)/3
*S* = 0.97	(Δ/σ)_max_ < 0.001
7401 reflections	Δρ_max_ = 0.31 e Å^−^^3^
546 parameters	Δρ_min_ = −0.22 e Å^−^^3^
7 restraints	Extinction correction: SHELXL97 (Sheldrick, 2008), Fc^*^=kFc[1+0.001xFc^2^λ^3^/sin(2θ)]^-1/4^
Primary atom site location: structure-invariant direct methods	Extinction coefficient: 0.00184 (13)

### Special details

**Table:**

Geometry. All esds (except the esd in the dihedral angle between two l.s. planes) are estimated using the full covariance matrix. The cell esds are taken into account individually in the estimation of esds in distances, angles and torsion angles; correlations between esds in cell parameters are only used when they are defined by crystal symmetry. An approximate (isotropic) treatment of cell esds is used for estimating esds involving l.s. planes.
Refinement. Refinement of F^2^ against ALL reflections. The weighted R-factor wR and goodness of fit S are based on F^2^, conventional R-factors R are based on F, with F set to zero for negative F^2^. The threshold expression of F^2^ > 2sigma(F^2^) is used only for calculating R-factors(gt) etc. and is not relevant to the choice of reflections for refinement. R-factors based on F^2^ are statistically about twice as large as those based on F, and R- factors based on ALL data will be even larger.

### Fractional atomic coordinates and isotropic or equivalent isotropic displacement parameters (Å^2^)

**Table:**

	*x*	*y*	*z*	*U*_iso_*/*U*_eq_	
O1	0.30526 (6)	0.71806 (9)	0.04126 (8)	0.0649 (4)	
O2	0.43048 (7)	0.88098 (9)	−0.07348 (7)	0.0650 (4)	
O3	0.19308 (8)	0.91363 (15)	0.23930 (9)	0.0919 (5)	
O4	0.10679 (6)	0.23802 (9)	0.22905 (6)	0.0524 (3)	
O5	0.03957 (6)	0.49125 (9)	0.15600 (7)	0.0621 (3)	
O6	−0.03644 (7)	−0.05271 (10)	0.09642 (8)	0.0709 (4)	
C1	0.35478 (8)	0.88485 (13)	0.02904 (10)	0.0504 (4)	
C2	0.39295 (9)	0.93766 (14)	−0.02605 (11)	0.0551 (4)	
C3	0.38780 (11)	1.04614 (15)	−0.03283 (13)	0.0708 (6)	
H3	0.4131	1.0819	−0.0703	0.085*	
C4	0.34683 (12)	1.09827 (16)	0.01425 (13)	0.0748 (6)	
H4	0.3440	1.1712	0.0088	0.090*	
C5	0.30795 (10)	1.05116 (15)	0.07093 (12)	0.0655 (5)	
C6	0.26594 (12)	1.10606 (18)	0.11951 (15)	0.0829 (7)	
H6	0.2635	1.1790	0.1139	0.099*	
C7	0.22837 (12)	1.06117 (19)	0.17422 (14)	0.0807 (7)	
H7	0.2002	1.1012	0.2064	0.097*	
C8	0.23233 (11)	0.95147 (18)	0.18221 (12)	0.0741 (5)	
C9	0.27245 (9)	0.89312 (15)	0.13665 (10)	0.0589 (5)	
H9	0.2746	0.8204	0.1438	0.071*	
C10	0.31102 (9)	0.94020 (14)	0.07859 (11)	0.0564 (4)	
C11	0.35854 (9)	0.77030 (13)	0.03250 (10)	0.0483 (4)	
C12	0.42958 (8)	0.71828 (12)	0.02502 (10)	0.0446 (4)	
C13	0.43721 (9)	0.65382 (13)	−0.04057 (9)	0.0475 (4)	
C14	0.37884 (10)	0.62973 (14)	−0.10021 (10)	0.0583 (5)	
H14	0.3324	0.6589	−0.0969	0.070*	
C15	0.38916 (12)	0.56528 (15)	−0.16185 (11)	0.0671 (5)	
H15	0.3496	0.5491	−0.2004	0.081*	
C16	0.45763 (12)	0.52246 (16)	−0.16906 (11)	0.0676 (5)	
H16	0.4636	0.4771	−0.2120	0.081*	
C17	0.51455 (11)	0.54500 (15)	−0.11592 (11)	0.0614 (5)	
H17	0.5605	0.5161	−0.1222	0.074*	
C18	0.50708 (9)	0.61207 (13)	−0.04985 (10)	0.0497 (4)	
C19	0.56517 (9)	0.63494 (13)	0.00600 (10)	0.0536 (4)	
H19	0.6118	0.6102	−0.0022	0.064*	
C20	0.55731 (9)	0.69286 (13)	0.07348 (10)	0.0499 (4)	
C21	0.61593 (10)	0.70988 (14)	0.13398 (12)	0.0618 (5)	
H21	0.6627	0.6852	0.1262	0.074*	
C22	0.60641 (11)	0.76003 (15)	0.20167 (12)	0.0706 (6)	
H22	0.6462	0.7701	0.2413	0.085*	
C23	0.53746 (11)	0.79769 (15)	0.21381 (11)	0.0668 (5)	
H23	0.5307	0.8308	0.2626	0.080*	
C24	0.48051 (9)	0.78739 (13)	0.15684 (10)	0.0549 (5)	
H24	0.4350	0.8158	0.1656	0.066*	
C25	0.48758 (8)	0.73474 (12)	0.08406 (10)	0.0450 (4)	
C26	0.47145 (10)	0.93094 (16)	−0.12941 (12)	0.0746 (6)	
H26A	0.5062	0.9787	−0.1011	0.090*	
H26B	0.4387	0.9693	−0.1682	0.090*	
H26C	0.4976	0.8790	−0.1572	0.090*	
C27	0.19236 (14)	0.8052 (2)	0.25240 (14)	0.1039 (8)	
H27A	0.2417	0.7812	0.2692	0.125*	
H27B	0.1736	0.7702	0.2030	0.125*	
H27C	0.1613	0.7895	0.2941	0.125*	
C28	0.01998 (8)	0.31643 (13)	0.13395 (9)	0.0427 (4)	
C29	−0.00418 (8)	0.41658 (14)	0.12093 (9)	0.0493 (4)	
C30	−0.07104 (9)	0.43797 (15)	0.07641 (11)	0.0598 (5)	
H30	−0.0869	0.5072	0.0679	0.072*	
C31	−0.11253 (9)	0.35791 (17)	0.04575 (10)	0.0615 (5)	
H31	−0.1572	0.3724	0.0148	0.074*	
C32	−0.09160 (8)	0.25468 (15)	0.05827 (10)	0.0511 (4)	
C33	−0.13472 (9)	0.17240 (18)	0.02490 (11)	0.0632 (5)	
H33	−0.1789	0.1872	−0.0069	0.076*	
C34	−0.11440 (9)	0.07300 (18)	0.03721 (11)	0.0642 (5)	
H34	−0.1436	0.0188	0.0134	0.077*	
C35	−0.04946 (9)	0.05029 (15)	0.08581 (11)	0.0560 (5)	
C36	−0.00565 (8)	0.12704 (14)	0.11868 (9)	0.0488 (4)	
H36	0.0381	0.1102	0.1506	0.059*	
C37	−0.02517 (8)	0.23192 (13)	0.10545 (9)	0.0453 (4)	
C38	0.09465 (8)	0.29910 (13)	0.17421 (9)	0.0417 (4)	
C39	0.15548 (7)	0.35787 (12)	0.14272 (9)	0.0392 (4)	
C40	0.19892 (8)	0.42532 (12)	0.19288 (9)	0.0423 (4)	
C41	0.19107 (9)	0.43916 (14)	0.27562 (10)	0.0545 (5)	
H41	0.1553	0.4010	0.2990	0.065*	
C42	0.23375 (10)	0.50569 (15)	0.32078 (11)	0.0647 (5)	
H42	0.2282	0.5122	0.3758	0.078*	
C43	0.28638 (10)	0.56567 (15)	0.28822 (11)	0.0635 (5)	
H43	0.3157	0.6122	0.3211	0.076*	
C44	0.29504 (9)	0.55677 (14)	0.20990 (10)	0.0559 (5)	
H44	0.3303	0.5978	0.1881	0.067*	
C45	0.25198 (8)	0.48675 (12)	0.15987 (9)	0.0441 (4)	
C46	0.25969 (8)	0.47903 (12)	0.07897 (9)	0.0451 (4)	
H46	0.2939	0.5218	0.0571	0.054*	
C47	0.21865 (8)	0.41046 (12)	0.02949 (9)	0.0399 (4)	
C48	0.22752 (8)	0.40196 (13)	−0.05310 (9)	0.0468 (4)	
H48	0.2602	0.4468	−0.0756	0.056*	
C49	0.19020 (8)	0.33104 (14)	−0.09977 (9)	0.0507 (4)	
H49	0.1968	0.3263	−0.1546	0.061*	
C50	0.14141 (8)	0.26408 (13)	−0.06721 (10)	0.0471 (4)	
H50	0.1167	0.2129	−0.1000	0.057*	
C51	0.12934 (8)	0.27177 (12)	0.01032 (9)	0.0425 (4)	
H51	0.0956	0.2266	0.0307	0.051*	
C52	0.16631 (7)	0.34640 (11)	0.06179 (9)	0.0377 (4)	
C53	0.01975 (11)	0.59695 (14)	0.14541 (13)	0.0739 (6)	
H53A	−0.0258	0.6095	0.1680	0.089*	
H53B	0.0135	0.6134	0.0884	0.089*	
H53C	0.0579	0.6406	0.1725	0.089*	
C54	0.02583 (11)	−0.08217 (15)	0.14738 (13)	0.0743 (6)	
H54A	0.0692	−0.0561	0.1259	0.089*	
H54B	0.0282	−0.1576	0.1509	0.089*	
H54C	0.0232	−0.0532	0.2006	0.089*	

### Atomic displacement parameters (Å^2^)

**Table:**

	*U*^11^	*U*^22^	*U*^33^	*U*^12^	*U*^13^	*U*^23^
O1	0.0497 (7)	0.0481 (8)	0.0986 (10)	−0.0031 (6)	0.0160 (6)	−0.0040 (7)
O2	0.0689 (8)	0.0554 (8)	0.0714 (8)	0.0007 (7)	0.0113 (7)	0.0108 (7)
O3	0.0797 (10)	0.1155 (14)	0.0815 (10)	0.0306 (10)	0.0133 (7)	−0.0171 (10)
O4	0.0528 (7)	0.0567 (8)	0.0466 (6)	−0.0016 (6)	0.0000 (5)	0.0105 (6)
O5	0.0507 (7)	0.0469 (8)	0.0862 (9)	0.0097 (6)	−0.0033 (6)	−0.0042 (6)
O6	0.0615 (8)	0.0577 (9)	0.0931 (10)	−0.0130 (7)	0.0064 (7)	−0.0037 (7)
C1	0.0421 (9)	0.0431 (11)	0.0622 (11)	0.0046 (7)	−0.0106 (7)	−0.0029 (8)
C2	0.0519 (10)	0.0444 (9)	0.0644 (11)	−0.0027 (8)	−0.0145 (8)	0.0071 (9)
C3	0.0748 (13)	0.0473 (9)	0.0867 (14)	0.0002 (11)	−0.0073 (11)	0.0039 (11)
C4	0.0799 (14)	0.0458 (12)	0.0927 (15)	0.0021 (11)	−0.0176 (11)	0.0017 (11)
C5	0.0575 (11)	0.0570 (13)	0.0759 (13)	0.0140 (9)	−0.0194 (8)	−0.0129 (9)
C6	0.0759 (14)	0.0768 (16)	0.0895 (16)	0.0238 (12)	−0.0191 (12)	−0.0249 (12)
C7	0.0665 (13)	0.0909 (13)	0.0795 (15)	0.0388 (13)	−0.0140 (11)	−0.0362 (13)
C8	0.0559 (11)	0.0943 (13)	0.0690 (12)	0.0231 (11)	−0.0064 (8)	−0.0194 (11)
C9	0.0476 (10)	0.0622 (13)	0.0636 (11)	0.0168 (9)	−0.0091 (8)	−0.0148 (9)
C10	0.0451 (9)	0.0512 (12)	0.0673 (11)	0.0077 (8)	−0.0180 (7)	−0.0071 (9)
C11	0.0436 (9)	0.0449 (11)	0.0547 (10)	−0.0011 (8)	−0.0016 (8)	0.0019 (8)
C12	0.0440 (9)	0.0386 (10)	0.0511 (9)	0.0005 (7)	0.0045 (7)	0.0058 (8)
C13	0.0540 (10)	0.0425 (10)	0.0458 (9)	−0.0050 (8)	0.0043 (8)	0.0057 (8)
C14	0.0630 (11)	0.0551 (12)	0.0554 (11)	−0.0042 (9)	0.0006 (9)	0.0037 (9)
C15	0.0831 (14)	0.0661 (14)	0.0499 (11)	−0.0146 (12)	−0.0027 (10)	0.0033 (10)
C16	0.0927 (16)	0.0626 (13)	0.0500 (11)	−0.0108 (12)	0.0181 (11)	−0.0042 (10)
C17	0.0749 (13)	0.0582 (12)	0.0546 (11)	−0.0043 (10)	0.0228 (10)	−0.0013 (10)
C18	0.0569 (10)	0.0462 (11)	0.0480 (10)	−0.0024 (8)	0.0146 (8)	0.0037 (8)
C19	0.0497 (10)	0.0526 (11)	0.0602 (11)	0.0027 (9)	0.0138 (8)	0.0003 (9)
C20	0.0463 (9)	0.0455 (10)	0.0577 (10)	0.0012 (8)	0.0053 (8)	0.0022 (9)
C21	0.0479 (10)	0.0583 (12)	0.0771 (13)	0.0071 (9)	−0.0028 (9)	−0.0041 (10)
C22	0.0633 (12)	0.0682 (14)	0.0748 (14)	0.0114 (11)	−0.0169 (10)	−0.0125 (11)
C23	0.0733 (13)	0.0635 (13)	0.0603 (11)	0.0150 (11)	−0.0070 (10)	−0.0131 (10)
C24	0.0542 (10)	0.0513 (11)	0.0585 (11)	0.0115 (9)	0.0027 (9)	−0.0043 (9)
C25	0.0452 (9)	0.0378 (10)	0.0519 (10)	0.0019 (7)	0.0043 (7)	0.0023 (8)
C26	0.0636 (12)	0.0801 (15)	0.0802 (14)	−0.0126 (11)	0.0081 (10)	0.0194 (12)
C27	0.0995 (19)	0.131 (3)	0.0864 (17)	0.0015 (18)	0.0328 (14)	−0.0037 (17)
C28	0.0360 (8)	0.0505 (11)	0.0423 (9)	0.0027 (8)	0.0074 (7)	0.0028 (8)
C29	0.0413 (9)	0.0532 (12)	0.0538 (10)	0.0047 (8)	0.0066 (8)	0.0006 (9)
C30	0.0466 (10)	0.0613 (13)	0.0708 (12)	0.0145 (9)	0.0029 (9)	0.0057 (10)
C31	0.0378 (9)	0.0843 (15)	0.0610 (11)	0.0096 (10)	−0.0002 (8)	0.0039 (11)
C32	0.0354 (8)	0.0676 (13)	0.0507 (10)	0.0008 (9)	0.0070 (7)	0.0005 (9)
C33	0.0403 (9)	0.0884 (16)	0.0601 (11)	−0.0058 (10)	0.0023 (8)	−0.0046 (11)
C34	0.0465 (10)	0.0803 (16)	0.0659 (12)	−0.0181 (10)	0.0065 (9)	−0.0113 (11)
C35	0.0487 (10)	0.0583 (13)	0.0626 (11)	−0.0070 (9)	0.0135 (8)	−0.0020 (9)
C36	0.0403 (9)	0.0555 (12)	0.0513 (10)	−0.0040 (8)	0.0075 (7)	−0.0002 (8)
C37	0.0356 (8)	0.0575 (12)	0.0439 (9)	−0.0011 (8)	0.0090 (7)	0.0022 (8)
C38	0.0413 (9)	0.0434 (10)	0.0407 (9)	0.0030 (7)	0.0054 (7)	−0.0006 (8)
C39	0.0337 (7)	0.0410 (9)	0.0424 (8)	0.0061 (7)	0.0021 (6)	0.0020 (7)
C40	0.0361 (8)	0.0475 (10)	0.0427 (9)	0.0067 (7)	0.0016 (7)	0.0000 (8)
C41	0.0502 (10)	0.0685 (13)	0.0443 (9)	0.0011 (9)	0.0031 (8)	−0.0048 (9)
C42	0.0596 (11)	0.0867 (15)	0.0463 (10)	0.0019 (11)	−0.0004 (9)	−0.0124 (10)
C43	0.0547 (11)	0.0728 (13)	0.0599 (12)	−0.0031 (10)	−0.0076 (9)	−0.0160 (10)
C44	0.0461 (9)	0.0594 (12)	0.0603 (11)	−0.0049 (9)	−0.0031 (8)	−0.0075 (9)
C45	0.0377 (8)	0.0462 (10)	0.0472 (9)	0.0024 (7)	−0.0010 (7)	−0.0028 (8)
C46	0.0368 (8)	0.0458 (10)	0.0530 (10)	−0.0016 (7)	0.0059 (7)	0.0017 (8)
C47	0.0341 (8)	0.0408 (9)	0.0444 (9)	0.0052 (7)	0.0031 (7)	0.0005 (7)
C48	0.0396 (8)	0.0560 (11)	0.0458 (9)	−0.0012 (8)	0.0085 (7)	0.0041 (8)
C49	0.0443 (9)	0.0664 (12)	0.0422 (9)	−0.0018 (9)	0.0075 (7)	−0.0054 (9)
C50	0.0399 (8)	0.0532 (11)	0.0483 (10)	−0.0018 (8)	0.0057 (7)	−0.0097 (8)
C51	0.0345 (8)	0.0442 (10)	0.0486 (9)	0.0010 (7)	0.0045 (7)	−0.0012 (8)
C52	0.0323 (7)	0.0382 (9)	0.0424 (9)	0.0055 (7)	0.0026 (6)	0.0015 (7)
C53	0.0721 (13)	0.0509 (13)	0.0974 (15)	0.0119 (10)	0.0038 (11)	0.0021 (11)
C54	0.0737 (14)	0.0528 (13)	0.0964 (15)	−0.0027 (11)	0.0094 (12)	0.0021 (11)

### Geometric parameters (Å, º)

**Table:**

O1—C11	1.2226 (18)	C26—H26B	0.9800
O2—C2	1.339 (2)	C26—H26C	0.9800
O2—C26	1.432 (2)	C27—H27A	0.9800
O3—C8	1.364 (2)	C27—H27B	0.9800
O3—C27	1.423 (3)	C27—H27C	0.9800
O4—C38	1.2203 (17)	C28—C29	1.383 (2)
O5—C29	1.3574 (19)	C28—C37	1.431 (2)
O5—C53	1.425 (2)	C28—C38	1.494 (2)
O6—C35	1.365 (2)	C29—C30	1.407 (2)
O6—C54	1.417 (2)	C30—C31	1.361 (3)
C1—C2	1.409 (2)	C30—H30	0.9500
C1—C10	1.424 (2)	C31—C32	1.403 (2)
C1—C11	1.487 (2)	C31—H31	0.9500
C2—C3	1.413 (3)	C32—C33	1.414 (2)
C3—C4	1.343 (3)	C32—C37	1.425 (2)
C3—H3	0.9500	C33—C34	1.352 (3)
C4—C5	1.402 (3)	C33—H33	0.9500
C4—H4	0.9500	C34—C35	1.414 (2)
C5—C6	1.390 (3)	C34—H34	0.9500
C5—C10	1.444 (3)	C35—C36	1.364 (2)
C6—C7	1.350 (3)	C36—C37	1.418 (2)
C6—H6	0.9500	C36—H36	0.9500
C7—C8	1.429 (3)	C38—C39	1.509 (2)
C7—H7	0.9500	C39—C40	1.408 (2)
C8—C9	1.359 (2)	C39—C52	1.4113 (19)
C9—C10	1.416 (2)	C40—C45	1.428 (2)
C9—H9	0.9500	C40—C41	1.432 (2)
C11—C12	1.501 (2)	C41—C42	1.350 (2)
C12—C25	1.402 (2)	C41—H41	0.9500
C12—C13	1.407 (2)	C42—C43	1.409 (3)
C13—C14	1.431 (2)	C42—H42	0.9500
C13—C18	1.432 (2)	C43—C44	1.355 (2)
C14—C15	1.365 (2)	C43—H43	0.9500
C14—H14	0.9500	C44—C45	1.424 (2)
C15—C16	1.407 (3)	C44—H44	0.9500
C15—H15	0.9500	C45—C46	1.393 (2)
C16—C17	1.342 (3)	C46—C47	1.389 (2)
C16—H16	0.9500	C46—H46	0.9500
C17—C18	1.433 (2)	C47—C48	1.427 (2)
C17—H17	0.9500	C47—C52	1.433 (2)
C18—C19	1.385 (2)	C48—C49	1.351 (2)
C19—C20	1.386 (2)	C48—H48	0.9500
C19—H19	0.9500	C49—C50	1.410 (2)
C20—C21	1.426 (2)	C49—H49	0.9500
C20—C25	1.435 (2)	C50—C51	1.357 (2)
C21—C22	1.344 (2)	C50—H50	0.9500
C21—H21	0.9500	C51—C52	1.425 (2)
C22—C23	1.408 (3)	C51—H51	0.9500
C22—H22	0.9500	C53—H53A	0.9800
C23—C24	1.356 (2)	C53—H53B	0.9800
C23—H23	0.9500	C53—H53C	0.9800
C24—C25	1.425 (2)	C54—H54A	0.9800
C24—H24	0.9500	C54—H54B	0.9800
C26—H26A	0.9800	C54—H54C	0.9800
			
C2—O2—C26	119.82 (15)	H27A—C27—H27C	109.5
C8—O3—C27	118.70 (17)	H27B—C27—H27C	109.5
C29—O5—C53	119.77 (14)	C29—C28—C37	119.79 (14)
C35—O6—C54	117.68 (14)	C29—C28—C38	118.86 (15)
C2—C1—C10	120.40 (16)	C37—C28—C38	121.28 (14)
C2—C1—C11	119.08 (16)	O5—C29—C28	115.55 (14)
C10—C1—C11	120.47 (16)	O5—C29—C30	122.95 (16)
O2—C2—C1	117.57 (15)	C28—C29—C30	121.47 (17)
O2—C2—C3	122.18 (17)	C31—C30—C29	118.89 (17)
C1—C2—C3	120.15 (18)	C31—C30—H30	120.6
C4—C3—C2	119.4 (2)	C29—C30—H30	120.6
C4—C3—H3	120.3	C30—C31—C32	122.25 (16)
C2—C3—H3	120.3	C30—C31—H31	118.9
C3—C4—C5	123.7 (2)	C32—C31—H31	118.9
C3—C4—H4	118.2	C31—C32—C33	121.58 (16)
C5—C4—H4	118.2	C31—C32—C37	119.39 (16)
C6—C5—C4	123.1 (2)	C33—C32—C37	119.03 (17)
C6—C5—C10	118.4 (2)	C34—C33—C32	121.39 (17)
C4—C5—C10	118.46 (18)	C34—C33—H33	119.3
C7—C6—C5	123.4 (2)	C32—C33—H33	119.3
C7—C6—H6	118.3	C33—C34—C35	119.60 (18)
C5—C6—H6	118.3	C33—C34—H34	120.2
C6—C7—C8	117.9 (2)	C35—C34—H34	120.2
C6—C7—H7	121.0	C36—C35—O6	124.78 (16)
C8—C7—H7	121.0	C36—C35—C34	121.17 (18)
C9—C8—O3	124.8 (2)	O6—C35—C34	114.05 (17)
C9—C8—C7	121.7 (2)	C35—C36—C37	120.26 (16)
O3—C8—C7	113.5 (2)	C35—C36—H36	119.9
C8—C9—C10	120.29 (19)	C37—C36—H36	119.9
C8—C9—H9	119.9	C36—C37—C32	118.52 (15)
C10—C9—H9	119.9	C36—C37—C28	123.39 (14)
C9—C10—C1	123.81 (17)	C32—C37—C28	118.02 (15)
C9—C10—C5	118.23 (17)	O4—C38—C28	121.83 (14)
C1—C10—C5	117.93 (17)	O4—C38—C39	120.82 (13)
O1—C11—C1	121.49 (15)	C28—C38—C39	117.33 (13)
O1—C11—C12	119.62 (15)	C40—C39—C52	120.94 (13)
C1—C11—C12	118.89 (14)	C40—C39—C38	120.26 (13)
C25—C12—C13	120.90 (14)	C52—C39—C38	118.78 (13)
C25—C12—C11	119.13 (14)	C39—C40—C45	119.09 (14)
C13—C12—C11	119.97 (14)	C39—C40—C41	123.44 (14)
C12—C13—C14	123.29 (15)	C45—C40—C41	117.42 (14)
C12—C13—C18	118.84 (15)	C42—C41—C40	120.96 (17)
C14—C13—C18	117.85 (15)	C42—C41—H41	119.5
C15—C14—C13	120.70 (18)	C40—C41—H41	119.5
C15—C14—H14	119.6	C41—C42—C43	121.50 (17)
C13—C14—H14	119.6	C41—C42—H42	119.3
C14—C15—C16	120.85 (18)	C43—C42—H42	119.3
C14—C15—H15	119.6	C44—C43—C42	119.81 (17)
C16—C15—H15	119.6	C44—C43—H43	120.1
C17—C16—C15	120.71 (18)	C42—C43—H43	120.1
C17—C16—H16	119.6	C43—C44—C45	120.90 (17)
C15—C16—H16	119.6	C43—C44—H44	119.5
C16—C17—C18	121.03 (19)	C45—C44—H44	119.5
C16—C17—H17	119.5	C46—C45—C44	121.03 (15)
C18—C17—H17	119.5	C46—C45—C40	119.57 (14)
C19—C18—C13	119.55 (15)	C44—C45—C40	119.39 (15)
C19—C18—C17	121.62 (16)	C47—C46—C45	121.78 (14)
C13—C18—C17	118.80 (16)	C47—C46—H46	119.1
C18—C19—C20	122.03 (16)	C45—C46—H46	119.1
C18—C19—H19	119.0	C46—C47—C48	121.48 (14)
C20—C19—H19	119.0	C46—C47—C52	119.53 (14)
C19—C20—C21	122.07 (16)	C48—C47—C52	118.99 (14)
C19—C20—C25	119.11 (15)	C49—C48—C47	121.00 (15)
C21—C20—C25	118.80 (16)	C49—C48—H48	119.5
C22—C21—C20	121.47 (17)	C47—C48—H48	119.5
C22—C21—H21	119.3	C48—C49—C50	120.14 (15)
C20—C21—H21	119.3	C48—C49—H49	119.9
C21—C22—C23	119.97 (18)	C50—C49—H49	119.9
C21—C22—H22	120.0	C51—C50—C49	120.90 (15)
C23—C22—H22	120.0	C51—C50—H50	119.6
C24—C23—C22	120.98 (18)	C49—C50—H50	119.6
C24—C23—H23	119.5	C50—C51—C52	121.23 (14)
C22—C23—H23	119.5	C50—C51—H51	119.4
C23—C24—C25	121.32 (16)	C52—C51—H51	119.4
C23—C24—H24	119.3	C39—C52—C51	123.45 (13)
C25—C24—H24	119.3	C39—C52—C47	118.93 (13)
C12—C25—C24	123.32 (14)	C51—C52—C47	117.59 (13)
C12—C25—C20	119.25 (15)	O5—C53—H53A	109.5
C24—C25—C20	117.36 (14)	O5—C53—H53B	109.5
O2—C26—H26A	109.5	H53A—C53—H53B	109.5
O2—C26—H26B	109.5	O5—C53—H53C	109.5
H26A—C26—H26B	109.5	H53A—C53—H53C	109.5
O2—C26—H26C	109.5	H53B—C53—H53C	109.5
H26A—C26—H26C	109.5	O6—C54—H54A	109.5
H26B—C26—H26C	109.5	O6—C54—H54B	109.5
O3—C27—H27A	109.5	H54A—C54—H54B	109.5
O3—C27—H27B	109.5	O6—C54—H54C	109.5
H27A—C27—H27B	109.5	H54A—C54—H54C	109.5
O3—C27—H27C	109.5	H54B—C54—H54C	109.5
			
C26—O2—C2—C1	−178.71 (14)	C53—O5—C29—C28	179.33 (15)
C26—O2—C2—C3	4.7 (2)	C53—O5—C29—C30	−2.8 (2)
C10—C1—C2—O2	−176.99 (14)	C37—C28—C29—O5	174.52 (13)
C11—C1—C2—O2	0.7 (2)	C38—C28—C29—O5	−8.5 (2)
C10—C1—C2—C3	−0.3 (2)	C37—C28—C29—C30	−3.4 (2)
C11—C1—C2—C3	177.33 (15)	C38—C28—C29—C30	173.60 (14)
O2—C2—C3—C4	177.20 (17)	O5—C29—C30—C31	−177.69 (16)
C1—C2—C3—C4	0.7 (3)	C28—C29—C30—C31	0.1 (3)
C2—C3—C4—C5	0.0 (3)	C29—C30—C31—C32	1.3 (3)
C3—C4—C5—C6	179.76 (19)	C30—C31—C32—C33	−178.92 (16)
C3—C4—C5—C10	−1.0 (3)	C30—C31—C32—C37	0.8 (3)
C4—C5—C6—C7	−179.99 (19)	C31—C32—C33—C34	−179.90 (16)
C10—C5—C6—C7	0.7 (3)	C37—C32—C33—C34	0.4 (2)
C5—C6—C7—C8	0.0 (3)	C32—C33—C34—C35	1.4 (3)
C27—O3—C8—C9	−1.0 (3)	C54—O6—C35—C36	2.2 (2)
C27—O3—C8—C7	179.54 (18)	C54—O6—C35—C34	−177.12 (16)
C6—C7—C8—C9	0.1 (3)	C33—C34—C35—C36	−2.0 (3)
C6—C7—C8—O3	179.52 (17)	C33—C34—C35—O6	177.34 (15)
O3—C8—C9—C10	179.69 (16)	O6—C35—C36—C37	−178.40 (14)
C7—C8—C9—C10	−0.9 (3)	C34—C35—C36—C37	0.9 (2)
C8—C9—C10—C1	179.38 (15)	C35—C36—C37—C32	0.8 (2)
C8—C9—C10—C5	1.6 (2)	C35—C36—C37—C28	−176.15 (14)
C2—C1—C10—C9	−178.42 (15)	C31—C32—C37—C36	178.80 (15)
C11—C1—C10—C9	4.0 (2)	C33—C32—C37—C36	−1.5 (2)
C2—C1—C10—C5	−0.7 (2)	C31—C32—C37—C28	−4.0 (2)
C11—C1—C10—C5	−178.28 (14)	C33—C32—C37—C28	175.70 (14)
C6—C5—C10—C9	−1.5 (2)	C29—C28—C37—C36	−177.69 (14)
C4—C5—C10—C9	179.16 (16)	C38—C28—C37—C36	5.4 (2)
C6—C5—C10—C1	−179.42 (16)	C29—C28—C37—C32	5.3 (2)
C4—C5—C10—C1	1.3 (2)	C38—C28—C37—C32	−171.61 (13)
C2—C1—C11—O1	−139.85 (17)	C29—C28—C38—O4	132.66 (16)
C10—C1—C11—O1	37.8 (2)	C37—C28—C38—O4	−50.4 (2)
C2—C1—C11—C12	40.1 (2)	C29—C28—C38—C39	−49.21 (19)
C10—C1—C11—C12	−142.21 (15)	C37—C28—C38—C39	127.73 (15)
O1—C11—C12—C25	−115.57 (18)	O4—C38—C39—C40	−61.2 (2)
C1—C11—C12—C25	64.4 (2)	C28—C38—C39—C40	120.68 (15)
O1—C11—C12—C13	64.3 (2)	O4—C38—C39—C52	120.84 (16)
C1—C11—C12—C13	−115.69 (17)	C28—C38—C39—C52	−57.30 (19)
C25—C12—C13—C14	176.42 (15)	C52—C39—C40—C45	3.4 (2)
C11—C12—C13—C14	−3.4 (2)	C38—C39—C40—C45	−174.59 (13)
C25—C12—C13—C18	−5.3 (2)	C52—C39—C40—C41	−179.19 (14)
C11—C12—C13—C18	174.81 (14)	C38—C39—C40—C41	2.9 (2)
C12—C13—C14—C15	−178.89 (16)	C39—C40—C41—C42	−179.32 (16)
C18—C13—C14—C15	2.8 (2)	C45—C40—C41—C42	−1.8 (2)
C13—C14—C15—C16	−1.1 (3)	C40—C41—C42—C43	1.5 (3)
C14—C15—C16—C17	−0.8 (3)	C41—C42—C43—C44	−0.2 (3)
C15—C16—C17—C18	0.8 (3)	C42—C43—C44—C45	−0.5 (3)
C12—C13—C18—C19	0.7 (2)	C43—C44—C45—C46	178.61 (16)
C14—C13—C18—C19	179.03 (15)	C43—C44—C45—C40	0.1 (2)
C12—C13—C18—C17	178.94 (14)	C39—C40—C45—C46	0.1 (2)
C14—C13—C18—C17	−2.7 (2)	C41—C40—C45—C46	−177.49 (14)
C16—C17—C18—C19	179.17 (17)	C39—C40—C45—C44	178.65 (14)
C16—C17—C18—C13	0.9 (3)	C41—C40—C45—C44	1.0 (2)
C13—C18—C19—C20	3.5 (3)	C44—C45—C46—C47	179.37 (15)
C17—C18—C19—C20	−174.67 (15)	C40—C45—C46—C47	−2.1 (2)
C18—C19—C20—C21	175.16 (16)	C45—C46—C47—C48	−179.13 (14)
C18—C19—C20—C25	−3.1 (2)	C45—C46—C47—C52	0.7 (2)
C19—C20—C21—C22	−175.28 (18)	C46—C47—C48—C49	176.31 (15)
C25—C20—C21—C22	3.0 (3)	C52—C47—C48—C49	−3.5 (2)
C20—C21—C22—C23	−0.4 (3)	C47—C48—C49—C50	0.2 (2)
C21—C22—C23—C24	−2.3 (3)	C48—C49—C50—C51	2.2 (2)
C22—C23—C24—C25	2.5 (3)	C49—C50—C51—C52	−1.1 (2)
C13—C12—C25—C24	−171.16 (15)	C40—C39—C52—C51	173.22 (14)
C11—C12—C25—C24	8.7 (2)	C38—C39—C52—C51	−8.8 (2)
C13—C12—C25—C20	5.8 (2)	C40—C39—C52—C47	−4.8 (2)
C11—C12—C25—C20	−174.35 (15)	C38—C39—C52—C47	173.18 (13)
C23—C24—C25—C12	177.12 (17)	C50—C51—C52—C39	179.70 (14)
C23—C24—C25—C20	0.1 (3)	C50—C51—C52—C47	−2.3 (2)
C19—C20—C25—C12	−1.6 (2)	C46—C47—C52—C39	2.8 (2)
C21—C20—C25—C12	−179.88 (15)	C48—C47—C52—C39	−177.42 (13)
C19—C20—C25—C24	175.54 (15)	C46—C47—C52—C51	−175.36 (13)
C21—C20—C25—C24	−2.8 (2)	C48—C47—C52—C51	4.5 (2)

### Hydrogen-bond geometry (Å, º)

*Cg*1, *Cg*2 and *Cg*3 are the centroids of the C20–C25, C1–C5/C10 and C47–C52 rings, respectively.

**Table:**

*D*—H···*A*	*D*—H	H···*A*	*D*···*A*	*D*—H···*A*
C7—H7···O4^i^	0.95	2.54	3.419 (3)	154
C46—H46···O1	0.95	2.57	3.2925 (19)	133
C49—H49···O4^ii^	0.95	2.57	3.2515 (19)	128
C50—H50···O6^iii^	0.95	2.56	3.368 (2)	143
C3—H3···*Cg*1^iv^	0.95	2.68	3.557 (2)	153
C26—H26*A*···*Cg*2^iv^	0.98	2.87	3.730 (2)	147
C30—H30···*Cg*3^v^	0.95	2.71	3.602 (2)	157

Symmetry codes: (i) *x*, *y*−1, *z*; (ii) *x*, −*y*+1/2, *z*−1/2; (iii) −*x*, −*y*, −*z*; (iv) −*x*+1, −*y*+2, −*z*; (v) −*x*, −*y*+1, −*z*.
